# Prognosis of Traumatic Injuries to the Anterior Teeth (Treated in Shahid Beheshti and Tehran Dental Schools During 1996-2001)

**Published:** 2006-04-01

**Authors:** Mohammad Asnaashari, Mohammad Amin Tavakkoli, Sara Shafiei Ardestani

**Affiliations:** 1 *Department of Endodontics, School of Dentistry, Shahid Beheshti University of Medical Sciences, Tehran, Iran*; 2 *Department of oral Radiology, School of Dentistry, Shahid Beheshti University of Medical Sciences, Tehran, Iran*; 3 *Pedodontis, Tehran, Iran*

**Keywords:** Anterior Teeth, Dental Trauma, Prognosis, Treatment

## Abstract

**INTRODUCTION:** Traumatic injuries to the teeth are among the most serious dental accidents, with the anterior teeth being mostly affected. Some consequences of dental trauma include misshaping, speech defects, psychological and social effects. The knowledge of the field can reduce the suffering, cost, and the time for patients, parents, and health care providers. The aim of this study was to investigate the treatment prognosis of anterior traumatized teeth in patients referred to Endodontics and pediatrics Departments of Shahid Behesthi and Tehran Dental Schools during 1996-2001.

**MATERIALS AND METHODS:** Fifty patients participated in this descriptive study. All affected by trauma to the teeth and completing the proposed treatment. Retrospective data, based on trauma forms as well as the clinical notes a questionnaire prepared for the study and analyzed in terms of age, gender, the type of trauma and etiology.

**RESULTS:** Eighty four percent of the studied traumatized teeth were maxillary centrals. Falling-outs are most frequent cause of the traumas (56.5%), followed by sport and play events (30.4%). Enamel- dentin fractures with and without pulpal involvement were the most prevalent trauma types. Most of the selected treatment procedures were involved with pulp and periapical areas.

**CONCLUSION:** Based on the finding of the study, the prognosis of traumatized anterior teeth in patients referred to the studied centers was estimated to be good.

## INTRODUCTION

Traumatic injuries to the teeth, in most cases cause the loss of all or a part of tooth structure. These injuries are common among children; with the maxillary anterior teeth being usually affected causing functional and esthetic problems. Some probable consequences of dental trauma could be misshaping, speech defects, psychological and social effects (1-3).

Andreasen (1990) has stated that all dental traumas must be considered as emergency cases and treated rapidly, so that, the prognosis will be improved by pain removal and correction of displaced teeth (4). However, the traumatized anterior tooth needs more attention. It is noteworthy that the traumatized teeth improve their chances of survival through careful evaluation and correct treatment done rapidly, unless complications such as teeth misshaping, pulp necrosis, apical radiolucencies, partial or total pulp calcification, root resorption, marginal periodontal bone loss, mainly happen. In some conditions the displacement of the teeth or tooth loss may even occur (5).

Zadik *et al.* (1979) studied the teeth, which had suffered from fracture of the enamel and dentin without pulp exposure to assess the period during which delayed pathologic changes may occur. They concluded that the most pathologic changes occur within the first 6 months following trauma. This finding necessitates the need for rapid management of traumatized teeth (6).

In an investigation done by Al Nazhan et al (1995), the complications arose from delayed management of traumatized permanent teeth were studied (7). It was found that most of the patients had a treatment delay exceeding 1 month. When the fracture involved both enamel and dentin, the frequency of pulp necrosis was 53%. The study also emphasized on the need for a proper diagnostic test to be done on the patients pulps and periodontal tissue comparing with initially nontreated dental injuries and to develop a trauma awareness educational program to encourage parents as well as the public to seek immediate dental treatment (7).

The need for appropriate (i.e. painless) treatment option in children and good knowledge about traumatology is stressed in the study performed by Robertson and Noren (1997).They also provided detailed information about adults who suffered from trauma since their childhood (8).

Accordingly, 39% of the patients reported dissatisfaction either with the color and/or anatomic form of the traumatized teeth or reconstruction. Most of the individuals did not remember having received any information about prognosis for the traumatized teeth. 21% of the patients remembered pain during treatment, and 25% remembered only the behavior and attitudes of the dental team (8).

Establishment of an appropriate rational treatment option mainly depends on careful diagnosis which can be developed through different methods of examinations. A complete history of the trauma would be achieved by asking a few standardized questions designed for this purpose (4).

Complete and detailed history of the trauma, as well as related clinical and radiographic assessments is needed for diagnosis. The history must include the time and the place of injury, its characteristics and the primary treatment done before referring to the clinic.

Etiology of dental injuries depends on different factors. Falling down was considered to be the primary causative factor. Other reasons are violence and fight, sport injuries, being struck, collisions and traffic accidents (9-12).

Etiological factors is important, since they can provide the basis of developing a preventive program by which trauma to teeth can be reduced to minimum. That is, when falling down is determined to be the main cause of trauma in early childhood, such trauma can be avoided by providing children and their parents with certain necessary information.

It should be considered that the prognosis of traumatized teeth is also different. Treatment delay, affected region, type and extent of trauma and developed treatment option are some determining factors of the prognosis (4). The study of prognosis in traumatized patients treated by scientifically treatment-based strategies can help investigators to manage and reduce complications after the dental trauma. It can also help to develop appropriate treatment and preventive plans in order to eliminate suffering, extra costs and waste of time for patients, parents, and health care providers (4).

This study investigated the treatment prognosis of anterior traumatized teeth in patients referred to Endodontic and Pediatric Departments of Shahid Beheshti and Tehran Universities, Dental Schools in Tehran -Iran during 1996-2001.

## MATERIALS AND METHODS

Descriptive study was based on data collected from fifty patients selected according to the following characteristics:

1. Enamel fracture, enamel dentin fracture with and without pulpal involvement, root fracture, and root and crown fracture.

2. A completed treatment strategy performed within at least 1-year follow up.

3. Available retrospective data in trauma forms, clinical notes and radiographies.

In the case of missing data, the patient was excluded. A questionnaire including patient's demographic information, trauma incidence time and cause, performed treatment procedures, fracture types, clinical (fistula, tooth mobility, color changes, pain, swelling, sensitivity to percussion/ palpation, periodontal/ periapical lesions, external/ internal root resorption, mineralization, and obturation) and radiographic squeal (periodontal ligament status, lamina durra axis, radiolucency, root pathologic recession and apex status) was prepared for the patients at the time they were referred and after the treatment.

**Table 1:** Frequency of traumatized teeth concerning the tooth location in patients referring to Pediatric and Endodontic Depts. of Shahid Beheshti and Tehran Dental Schools during 1996-2001.

**Tooth location**	**Frequency**	**Percentage**
Right central maxillary	16	32%
Right lateral maxillary	2	4%
Right central mandibular	2	4%
Left central maxillary	21	42%
Left lateral maxillary	2	4%
Left central mandibular	1	2%
Left & right central maxillary	5	10%
Left & right central mandibular	1	2%
**Total**	**50**	**100%**

All records of the referred traumatic patients were examined and fifty records were finally selected, while the remaining 150 cases were excluded for not meeting the requirements of the study. The questionnaires were filled using the data in the records and analyzed in terms of age, gender, type and etiology of the trauma.

For statistical analysis, clinical and radiographic symptoms were given an equal numerical value of one. Therefore, the sum of the values presented an index for the success rate of treatment procedure selected for the patients, i.e. in a patient with fistula, tooth color change, no mobility, pain and swelling, the index calculated to be 2 (1+1+0+0+0). The same method was used for radiographic symptoms. The collected data were analyzed by means of SPSS statistical software.

## RESULTS

The age average of the samples was 11 years and 10 months having a standard deviation of 5 years and 1 month. 48% of the patients (24 cases) were girls and 52% of them (26 cases) were boys. The average time interval of referral for treatment was 1 year after the incidence of trauma with standard deviation of 5.6 months.

21 cases (42%) showed traumatized left maxillary central teeth while the right maxillary centrals had the next rank with the incidence rate of 32% among overall traumas (16 cases). In addition, 5 cases showed trauma to both left and right upper teeth. Therefore, 84% of all traumatic case were anterior teeth including maxillary centrals (Table 1).

**Figure 1:**
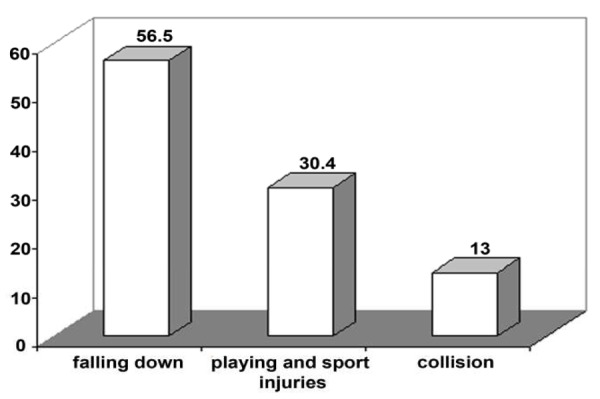
Etiology of trauma in patients referring to Pediatric and Endodontic Depts. of Shahid Beheshti and Tehran Universities, Dental Schools for trauma causing factors during 1996-2001.

Among 46 patients reporting the cause of trauma, falling down had been reported as the most frequent etiological factor (26 patients), playing and sport injuries as the second most frequent factor (14 cases), followed by collision occurring in 6 patients (Figure 1).

According to the data collected from patients' records about the trauma form, enamel– dentin fracture without pulpal involvement was the most common type of injury as reported in 21 cases. Enamel dentin fracture with pulpal involvement, root fracture and crown and root fracture had the next rank (20,8, and 1 case, respectively).

Pulp and periapical treatments as performed in 38 traumatized teeth were observed more frequently in patients, 7 cases received restoration and follow up treatment and 4 cases had follow up treatment only.

The received treatment procedure had completely removed fistula, mobility, pain, PDL status, lamina dura axis, radiolucency and root pathologic recession among clinical and radiographic sequelae of the traumatized teeth. The treatment techniques developed to restore the fractured teeth with original color could not result in significant improvements. The same outcome happened about the apex status among studied radiographic squeal (Table 2).

## DISCUSSION

The study revealed the average treatment delay of 1 year among traumatized patients referred to the study centers. As stated by Al Nazhan (1995), treatment delay exceeding 1 month may result in pulpal pathologic injuries (7), the received treatment strategies are considered as delayed managements. There is no doubt that immediate emergency repairs could have produced better treatment outcomes. The minimum time of 1 year for treatment follow up was considered as inclusion criteria, so that the groups whose records are not filed for a long time were exclude, therefore the results were only related to the patients with this follow up period.

**Table 2:** Clinical and radiographic sequelae of traumatized teeth at the time of injury and after the treatment in patients referred to Pediatric and Endodontic Depts. of Shahid Beheshti and Tehran Universities, Dental Schools for trauma causing factors during 1996-2001.

**S** **e** **qu** **e** **la** **e**		**Injury ** **time**	**After the ** **tr** **eat** **m** **e** **n** **t**	**Ch** **a** **n** **ges**
Clinical	Fistula	18%	0%	18%
	Mobility	38%	0%	38%
	Discoloration	10%	4%	6%
	Pain	56%	0%	56%
	Swelling	10%	0%	10%
	POL status	58%	0%	58%
Radiographic	Lamina dura axis	28%	0%	28%
	Radiolucency	38%	0%	38%
	Root pathologic recession	12%	0%	12%
	Apex status	54%	14%	40%

No enamel fracture was observed in the traumatized teeth used in this study. The findings may be due to the control of these fractures by the family dentists without the need for further examinations at dental hospitals. It must be declared that most of the patients under study had received initial emergency treatments at different dental clinics or had referred to the dental schools by the dentists. So, the outcome of these primary procedures must be considered when the final results are interpreted.

Robertson et al (2000) stated that pulp healing was related to the following clinical factors: stage of root development at the time of injury, associated damage to the periodontium at the time of injury (luxation) and the time interval between injury and the initial treatment (13).

Most of the patients with no pulpal exposure had received restorative and follow up treatment option. Zerman et al (1990) studied the diagnosis and treatment of traumatic root fractures and emphasized that endodontic therapy should not be performed unless there is evidence of a pulp exposure (14). Their direction in choosing treatment procedure is considered in the present study.

Cavalleri and Zerman (1995), Saroglu and Sonmaz (2002), and Zadik (1976) have reported fracture of enamel and dentine without pulpal exposure to be the most common type of trauma (80%, 50.5%, 55%) in the study of traumatic crown fractures in permanent incisors, treated in the pedodontic clinic of Ankara University, Turkey, during 18 months, and a survey of traumatized primary anterior teeth in Jerusalem preschool children (15,16,17). Although with different incidences, the present study also indicated the same findings about trauma type (42%). Jacobsen (1980) has reported subluxation as the most common type of injury in the study of traumatized permanent incisors (18).

The present study showed left and right central maxillary teeth as the most frequently affected teeth (42% and 32% respectively). In the study of assessing the prevalence and distribution of the traumatic injuries to the anterior teeth among school children from South Kanara, India, the maxillary central incisors were commonly affected in both primary and permanent dentition (19). The maxillary anterior teeth represented 96.1% of the cases with traumatized permanent teeth in Brazilian children, while the central incisor teeth were the most affected as reported by Rocha and Cardoso (2001) (20). The maxillary incisors accounted for 96% of the fractured teeth among Texas and Harris County children as well (21).

As stated previously, the primary causative factor in the occurrence of trauma was falling down (56.5%), followed by playing and sport accidents and collision (foreign body strike). Falling down has been shown to be the most frequent etiology of trauma as observed in 83.3% of cases by Rocha and Cardoso (2001), 63% by Wilson et al (1997), 27.7% by Canakci et al (2003), and 67.34% by Saroglu Sonmez (2002) (12,16,20,22).

The prevalence of traumatized permanent anterior teeth was assessed among patients referred to Shahid Beheshti Medical University, Dental School during 1999-2000 (23). In this study the majority of traumas were related to the maxillary central teeth and falling down was the cause for 40% of trauma cases. 18% of the cases were caused by sport and 13.3% of cases were caused by playing accidents and collision.

All clinical symptoms of the studied traumatized anterior teeth except discoloration and apex development status had undergone significant improvement.
